# Use of Health Apps and Wearable Devices: Survey Among Italian Associations for Patient Advocacy

**DOI:** 10.2196/10242

**Published:** 2019-01-15

**Authors:** Paola Mosconi, Silvia Radrezza, Emanuele Lettieri, Eugenio Santoro

**Affiliations:** 1 Laboratory for Medical Research and Consumer Involvement Department of Public Health Istituto di Ricerche Farmacologiche Mario Negri IRCCS Milan Italy; 2 Department of Management, Economics and Industrial Engineering Politecnico of Milan Milan Italy; 3 Laboratory of Medical Informatics Department of Public Health Istituto di Ricerche Farmacologiche Mario Negri IRCCS Milan Italy

**Keywords:** health apps, mobile phone, Web-based survey, patients’ health care advocacy associations, wearable devices

## Abstract

**Background:**

Technological tools such as Web-based social networks, telemedicine, apps, or wearable devices are becoming more widespread in health care like elsewhere. Although patients are the main users, for example, to monitor symptoms and clinical parameters or to communicate with the doctor, their perspective is seldom analyzed, and to the best of our knowledge, no one has focused on the patients’ health care advocacy associations’ point of view.

**Objective:**

The objective of this study was to assess patients’ health care advocacy associations’ opinions about the use, usefulness, obstacles, negative aspects, and impact of health apps and wearable devices through a Web-based survey.

**Methods:**

We conducted a Web-based survey through SurveyMonkey over nearly 3 months. Participants were contacted via an email explaining the aims of the survey and providing a link to complete the Web-based questionnaire. All the 20 items were mandatory, and the anonymized data were collected automatically into a database. Only fully completed questionnaires were considered for analysis.

**Results:**

We contacted 1998 patients’ health care advocacy associations; a total of 258 questionnaires were received back (response rate 12.91%), and 227 of the received questionnaires were fully completed (completion rate 88.0%). Informative apps, hospital apps for viewing medical reports or booking visits, and those for monitoring physical activity are the most used. They are considered especially useful to improve patients’ engagement and compliance with treatment. Wearable devices to check physical activity and glycemia are the most widespread considering, again, their benefits in increasing patients’ involvement and treatment compliance. For health apps and wearable devices, the main obstacles to their use are personal and technical reasons; the risk of overmedicalization is considered the most negative aspect of their constant use, while privacy and confidentiality of data are not rated a limitation. No statistical difference was found on stratifying the answers by responders’ technological level (*P*=.30), age (*P*=.10), and the composition of the association’s advisory board (*P*=.15).

**Conclusions:**

According to responders, health apps and wearable devices are sufficiently known and used and are considered potential supports for greater involvement in health management. However, there are still obstacles to their adoption, and the developers need to work to make them more accessible and more useful. The involvement of patients and their associations in planning services and products based on these technologies (as well as others) would be desirable to overcome these barriers and boost awareness about privacy and the confidentiality of data.

## Introduction

New technologies, connectivity, and availability of the internet have boosted the development of mobile apps and wearable devices in the last decade. Those for health and care focus mainly on the management of chronic conditions such as diabetes [1], cardiovascular diseases [2], and specific populations or conditions [3,4]. Moreover, these devices have gained ground in health self-monitoring and preventive medicine [5]. Italy is the second most frequent worldwide user of new technology, particularly wearables, after the United States and before Germany and France [6].

Electronic health (eHealth) and medical devices such as apps and wearable devices are becoming more and more important in health debate. In the past year alone, health apps have grown by nearly 78,000 units, reaching a total of 325,000 registered [7]*.* Despite their spread, however, the use of these new technologies arouses discussions about the collection and sharing of data, focused on their protection, privacy, accuracy, and reliability [8,9].

A recent study about the most downloaded “mobile health (mHealth) apps” found that only 30.5% had a privacy policy. However, many of these documents used technical language not accessible to most lay people or they did not focus enough on the app itself [10,11]. Moreover, health data are often stored in the “cloud” so we cannot know where it is exactly, making it not completely safe [12].

There is still debate in the literature on the use and efficacy of apps and wearable devices to help patients collect and access their clinical data [13]. To date, patients’ engagement is considered an important clinical outcome, and these tools could make them more responsible for treatment management, monitoring symptoms, identifying risk factors, and how best to prevent other diseases, for example, by changing their lifestyle [14]. Health apps or wearable devices could be suitable above all for chronic diseases. Evidence of this comes from a systematic review based on randomized controlled trials, where Yuan et al reported that the use of mobile apps by diabetic adults lowered glycemia more than standard care alone [15].

Several surveys on consumer perspective [16,17], use of mHealth app [18], and wearable devices [19] have been conducted regarding the use of technological tools in health care; however, to the best of our knowledge, none has been related to patients’ health care advocacy associations. These associations, in Italy, like in other industrialized countries, are a growing reference point in the public health debate and innovation, influencing research and the political agenda [20]. The associations are spokespersons for a multiplicity of opinions and collecting their points of view is the best way to actually engage consumers and patients better. We conducted an observational study through a Web-based survey to analyze the representatives of health care advocacy associations’ point of view.

## Methods

### Methodology

The Web-based survey was close, voluntary, and took only a few minutes to complete. No incentive was given. The SurveyMonkey Web-based survey service [21] was used to design the questionnaire, manage the survey, and collect data. The Web questionnaire was 5 pages long, and all data were anonymized and protected by Norton and TRUSTe.

An email was sent to 1998 contacts, describing the survey and including a link inviting them to complete the questionnaire. Reminder emails were sent after 3, 5, and 7 weeks. The questionnaire was organized in a series of linked pages (multiple-item screens) with electronic instructions to facilitate the flow. A progress indicator was permanently visible, and compilers could enter a personal comment for each question. The questions were in bold type, and the answers had optional buttons.

According to the “Checklist for Reporting Results of Internet E-Surveys” (CHERRIES), all the questions, except the last one, were mandatory to obtain more solid data on the endpoints of the survey. To guarantee the possibility of answering, most questions provided a nonresponse option, that is, “I don’t know.” Again, based on the CHERRIES checklist, we analyzed only completed questionnaires, excluding questionnaires that had missing data due to the responder stopping early and leaving the website.

If all answers were not completed, it was not possible to continue and confirm the questionnaire. A “back button” was provided to change answers before submitting them, but thereafter, no further changes were allowed. The answers were collected automatically in the SurveyMonkey database. Overall, 6 questionnaires were filled manually in the database by the coordinator center to clean up any technical problems of the responders. Of note, under Italian law, ethical approval was not required for this kind of survey.

### Development of the Questionnaire

We conducted a literature review in PubMed using the following keywords: “digital technologies,” “survey,” “questionnaire,” “eHealth,” “mHealth,” “digital health,” “digital innovation,” and “development.” We retrieved a number of surveys about “digital innovation” involving citizens but none involved patients’ health care advocacy associations. Based on the material collected, we drafted 20 items about the use, usefulness, obstacles, negative aspects, and impact related to eHealth technologies, focusing on health apps and wearable devices.

In addition, we recorded the characteristics of the patients’ health care advocacy associations contacted (year of foundation, setting, geographic distribution, and current composition of the advisory board) and of responders (sex, age, education, personal use of health apps, or wearable devices) to have a complete framework of the sample (see Multimedia Appendix 1). In this regard, some previous studies showed that age and individual technological level influenced a person’s interest in the adoption of health apps also in clinical situations [22].

We pretested the questionnaire with the collaboration of 16 representatives of patients’ health care advocacy associations to assess its readability, clarity, and completeness and to collect suggestions. Seven representatives answered, and a general positive consensus was gathered; hence, no major changes were required.

### Recruitment

We started from a database of patients’ health care advocacy associations available at the “Laboratory for Medical Research and Consumer Involvement” (n=2087); duplicate and wrong email addresses were removed (n=98). This database, developed in 2004, includes contacts of members of the board of associations—no single member—who had participated in previous research projects, training courses, and initiatives.

We sent one email invitation to each association, usually to the president of the association or a member of the board. The questionnaire asked for his or her personal opinion (see questions 7-10 and 14 in the Multimedia Appendix 1) and to report the common belief of the members represented (see questions 11-13 in the Multimedia Appendix 1).

The survey was announced through Web on the Mario Negri Institute websites and 2 patients’ health care association websites; 9 health care associations requested to participate by contacting the coordinator center directly. Overall, 1998 patients’ health care advocacy associations were reached via emails. A unique site visitor was permitted by checking the internet protocol address of responder. Results were presented according to CHERRIES” [23] and a similar guideline formulated by Bennett et al [24].

### Statistical Analysis

Descriptive analyses were conducted using SAS version 9.4 (SAS Institute Inc) to cross variables according to several criteria. On the basis of the previous studies suggestions, we analyzed the pattern of answers by responders’ age and their technological level. In addition, we considered the composition of the advisory board of the patients’ health care advocacy associations as a possible confounding factor. A statistical test (*P* value) was expected only for results that gave significant patterns using a predetermined alpha level of .05.

## Results

The survey was open from March 23 to June 8, 2017. A total of 258 answers were collected, giving a response rate of 12.91% (258/1998). We analyzed 227 completed questionnaires, as 31 responders dropped out before completing the questionnaire, giving a completion rate of 88.0% (227/258).

Tables 1 and 2 summarize the main characteristics of the patients’ health care advocacy associations and responders, respectively. Out of 227 responders, 121 (53.3%) worked locally, while 43 (18.9%) and 63 (27.8%) worked on the regional and national levels, respectively. Most associations were based in the north of Italy. The advisory board comprised all or most of the patients or relatives for 58.6% (133/227) answers; furthermore, 18.1% (41/227) responders stated that there were no patients or relatives on their board. Respondents were aged 25-88 (mean age 56, SD 12) years; females and males were fairly represented, and 46.3% (105/227) respondents reported an appreciable level of technology, using at least one health app or wearable device, or both.

According to the 227 participants, Web-based social networks were used for health communication, promotion, or grouping patients with a specific disease, and telemedicine had a great impact on health care out of all technological innovations, with 183 (80.6%) responders and 178 (78.4%) of preferences, respectively; these were followed by wearable devices, such as smart-watches or wristbands, and health apps, with 148 (65.2%) and 146 (64.3%) responders, respectively. However, they foresaw that in the next 3 years, the impact would increase more for health apps, wearable devices, and telemedicine services than for Web-based social networks (Table 3).

Among health apps, those providing health and disease information, hospital apps for services such as viewing medical reports or booking visits, and those for tracking fitness or physical activity were the most used by members of health care advocacy associations, with 149 (65.6%), 118 (52.0%), and 116 (51.1%) of 227 participants, respectively. Apps to improve treatment and medication compliance, health or condition trackers, and diet or nutrition apps followed with 106 (46.7%), 96 (42.2%), and 80 (35.2%) of 227 responders, respectively. Symptom-checker apps for self-diagnosis were the least used, with 22.0% (50/227) positive answers. Wearable devices monitoring physical activity and glycemia were the most widespread among 227 respondents—110 (48.5%) and 108 (47.6%), respectively—followed by those monitoring heart rate and blood pressure, weight, and sleep tracking, with 106 (46.7%), 88 (38.8%), and 64 (28.2%) respondents, respectively.

From the point of view of patients’ health care advocacy associations’ representatives, health apps and wearable devices are not only useful to improve patients’ engagement in their own health and treatment compliance but also to help them understand their health status and conditions, enhancing the communication between patients and physicians, and reducing health care costs (Table 4).

**Table 1:** Characteristics of patients’ health care advocacy associations (N=227).

Characteristics		Associations, n (%)	
**Year of foundation**			
	1940-1999		119 (52.4)
	2000-2016		108 (47.6)
**Types**			
	Oncology		38 (16.7)
	Diabetes		34 (15.0)
	Rare diseases		28 (12.3)
	Neurology		19 (8.4)
	Cardiovascular		15 (6.6)
	Disability		12 (5.3)
	Breast cancer		13 (5.7)
	Pediatric		7 (3.1)
	AIDS		5 (2.2)
	Brain-injured		4 (1.8)
	Autism		2 (0.9)
	Asthma		1 (0.4)
	Other		49 (21.6)
**Geographic distribution**			
	North		126 (55.5)
	Center		50 (22.0)
	South-Islands		51 (22.5)
**Weblink**			
	Website		203 (89.4)
	Facebook account		186 (81.9)
	Twitter account		80 (35.2)
	YouTube channel		74 (32.6)
	Blog		43 (18.9)
**Advisory board**			
	All members are patients or their relatives		78 (34.4)
	Most members are patients		55 (24.2)
	Patients’ representatives are nearly half		27 (11.9)
	Patients’ representatives are a minority		26 (11.5)
	There are no patients’ representatives		41 (18.1)

**Table 2:** Characteristics of responders (N=227).

Characteristics			Responders, n (%)
**Sex**			
	Males	100 (44.1)	
	Females	127 (56.0)	
**Age in years**			
	≤50	67 (29.5)	
	51-65	106 (46.7)	
	>65	54 (23.8)	
**Education level**			
	Elementary school	1 (0.4)	
	Secondary school	10 (4.4)	
	High school	93 (41.0)	
	Degree or superior	121 (53.3)	
	Other	2 (0.9)	
**Personal use of health app or wearable**			
	Both	38 (16.7)	
	Only health app	50 (22.0)	
	Only wearable	17 (7.5)	
	None	122 (53.7)	

**Table 3:** Impact of technological tools on medical care and health (N=227).

Technological tool	Responders, n (%)				
	Current impact			Future impact	
	Yes	No	Yes		No
Health app	146 (64.3)	81 (35.7)	192 (84.6)		35 (15.4)
Wearable	148 (65.2)	79 (34.8)	183 (80.6)		44 (19.4)
Telemedicine	178 (78.4)	49 (21.6)	211 (93.0)		16 (7.1)
Social network	183 (80.6)	44 (19.4)	197 (86.8)		30 (13.2)

**Table 4:** Utility of health apps and wearables in health care (N=227).

Utility	Responders, n (%)	
	Positive effect	Negative or no effect
Empowerment in own health	204 (89.9)	23 (10.1)
Improve doctor-patient communication	167 (73.6)	60 (26.4)
Understand own health condition	179 (78.9)	48 (21.1)
Reduce public health costs	127 (55.9)	100 (44.1)
Improve compliance	186 (81.9)	41 (18.1)

Responders reported that the main obstacles to the adoption of health apps and wearable devices among members of their health care advocacy associations were personal motivations, such as concern about not being able to use them, or technical reasons, including owning an unsuitable smartphone, followed by the lack of evidence of their usefulness, accuracy, and reliability. Conversely, the lack of confidence in data protection and confidentiality seemed to restrict their use for only 31.3% (71/227) responders. It was interesting to highlight that about a quarter of responders had no clear opinion about the privacy and accuracy of data collected through apps and wearable devices (Table 5).

**Table 5:** Obstacles related to the use of health apps and wearables (N=227).

Obstacles	Yes, n (%)	No, n (%)	I don’t know, n (%)
Technical barrier	144 (63.4)	52 (22.9)	31 (13.7)
Personal opinion	144 (63.4)	48 (21.2)	35 (15.4)
Low trust in recorded data utility	73 (32.2)	89 (39.2)	65 (28.6)
Low trust in data reservation and privacy	70 (30.8)	100 (44.1)	57 (25.1)
Low trust in recorded data reliability and quality	71 (31.3)	80 (35.2)	76 (33.5)
Low example of their utility in public health	90 (39.6)	59 (26.0)	78 (34.4)

**Table 6:** Negative aspects of constant adoption of health apps and wearable devices (N=227).

Negative aspects	Yes, n (%)	No, n (%)
Become dependent	148 (65.2)	79 (34.8)
No privacy	70 (30.8)	157 (69.2)
Excessive control of own health	149 (65.6)	78 (34.4)
Overmedicalization	160 (70.5)	67 (29.5)
Weaken doctor-patient communication	101 (44.5)	126 (55.5)

Among negative aspects or risks about the use of health apps and wearable devices, respondents considered first overmedicalization (ie, the overuse of drugs, supplements, and medical devices or scheduling medical examinations even if they are not really needed), followed by the risk of becoming dependent on technology, or weakening patient-doctor communication (Table 6). The lack of confidence in the protection and confidentiality of data was considered a negative aspect only by 30.8% (70/227) responders in agreement with the answers related to the obstacles reported in Table 5.

When we asked about the kind of health apps on which developers should focus in future to improve medical care and health, respondents put first those improving and disseminating health services and tools, with out of 227, 186 (81.9%) preferences, followed by those boosting compliance (175, 77.1%) and those monitoring vital signs (156, 68.7%), diet and nutrition (147, 64.8%), and physical activity (137, 60.4%). Of the 227 respondents, about two-third (154, 67.8%) asked to focus the efforts on informative health apps, while only 101 (44.5%) wanted more attention to symptom-checker apps. For wearable devices, the respondents stated that more attention could be paid to heart rate and blood pressure monitoring (167, 73.4%), glycemia (165, 72.7%), weight monitoring (139, 61.2%), and fitness trackers (136/227, 60.0%); less interest was shown in sleep trackers (112, 49.3%).

Data about the use, usefulness for health care, obstacles, and negative aspects of health apps and wearable devices were stratified by the respondents’ age (≥58 years), technological level (users of one health app or one wearable device at least compared to not users), and the composition of the association’s advisory board (all or most are patients compared to few or no patients). There were no statistically significant associations, although there was a slight influence of the technological level of responders. High technological level responders reported a greater positive impact of all technological tools (health apps, wearable devices, telemedicine, and Web-based social networks) on health and health care, now and in the future than the lower technological level responders. In addition, attitudes were different toward the usefulness of health apps and wearable devices. According to high technological responders, patients’ engagement and compliance with treatment related to these tools were most important. In addition, higher technological level responders considered the risk of becoming dependent and excessive control of one’s own health less important than the less technological ones.

## Discussion

This study offers an exclusive view of patients’ health care advocacy associations’ opinions about eHealth technological tools that have not yet been well explored. The results suggest that the most commonly used health apps appear to be informative apps, apps providing access to hospital services, and fitness or physical activity tracking apps, while the favorite wearable devices are those for fitness, blood glucose, and heart rate monitoring. Our findings are different from those reported in a recent study based on citizens where fitness, diet or nutrition, and symptom navigator apps were at the top of the rankings with 59%, 52%, and 36% of use, respectively [17]. In addition, our results differ from those reported in a recent study focused on consumer’s perceived attitudes about wearable devices in health monitoring, in which exercise coaching (61%) and location tracking (59%) came first [19].

Despite this, our results are similar to those from a study conducted on patients and confirm how both health apps and wearable devices can be used for patient engagement. In fact, a recent survey found that the main reasons for their adoption were chronic disease management (81%), support for medical adherence (66%), and fitness tracking (46%) [13].

Respondents are optimistic about the future of health apps and wearable devices. They suggest that in the near future, developers should focus on apps providing more services, increasing drug and therapy compliance, and monitoring vital signs.

The gaps between current opinion and needs may be explained considering that today’s responders are concerned about the reliability of the data collected by health apps and wearable devices but are confident that in the future, more evidence will be provided to support their use and efficacy in collecting data for diseases monitoring and management. On the other hand, our survey shows that the lack of evidence of reliability and accuracy of health apps and wearable devices, along with difficulty in adopting them, are the main obstacles to their use.

About the accuracy—but also the effectiveness—of these tools, researchers need to raise the overall quality of interventional trials conducted on patients, focusing on mobile apps and wearable devices. These trials are still limited due to the short follow-up, low recruitment rate, and high proportion of withdrawals before the scheduled time. It is, therefore, hard to see whether they might become valuable for helping patients with their own health [25].

Other studies raised the question of discontinuing the use of health apps and wearable devices when the real setting is not taken into consideration. For example, a recent study showed that the majority of users increased their physical activity after purchasing a wearable device, but nearly one-third stopped tracking after just 6 months [26]. Another study found that 55% of the 325,000 health apps available in the app stores are downloaded <5000 times and only 2% of all health apps count >500,000 monthly active users [7]. Fuller involvement of patients and their associations to identify specific needs could probably narrow this gap and make wearable devices and health apps more appealing and useful for engagement [27].

The lack of confidence in data protection and confidentiality seemed not to limit the use of health apps and wearable devices and did not appear to be one of the main negative aspects. This is very important because the adoption of apps and wearable devices in health care might give rise to challenges about security, data protection, and data reuse [8,9]. For example, a study on 79 apps certified as clinically safe and trustworthy by the “UK-National Health Service Health Apps Library” revealed that 89% of them transmitted information to Web-based services, and none encrypted personal information stored locally. Two-thirds of the apps sent personal information over the internet without encryption and 20% did not have a specific privacy policy [28]. However, it is interesting that about a quarter of our responders knew little about the question of data privacy and confidentiality; a possible explanation is that patients’ representatives themselves do not know enough, necessitating more awareness and information. An alternative reason is that participants do not care much about these topics and are willing to exchange part of their data for a potential health care gain.

This survey has several limitations. First, respondents are representatives of health care advocacy associations and answered reporting their point of view and the patients’ perspective. Second, as Italy has no central database of consumers’ and patients’ associations, we contacted a limited number of associations; hence, this sample may not be representative of all Italian situations and attitudes. The response rate, 12.91% (258/1998), may have increased the selection bias, but it agrees with other similar studies. The response rate of Web-based surveys is often low, and many strategies have been investigated to increase it [29]. Finally, the heterogeneity of the health care advocacy associations that participated could have added further selection bias.

In conclusion, the survey shows that health apps and wearable devices are sufficiently used and appreciated by patients as potential supports for greater engagement in their health. There are still obstacles to their use, however, on which developers should work to make them more accessible and more useful. The involvement of patients and their health care advocacy associations in designing services and products based on these technologies—and others—is desirable to overcome barriers and make their development and acceptance easier and more competitive.

## Appendix

Multimedia Appendix 1 Questionnaire provided to participants (English version).

## Abbreviations

CHERRIES: Checklist for Reporting Results of Internet E-Surveys
eHealth: electronic health
mHealth: mobile health
