# Supplementary material for: Use of Health Apps and Wearable Devices: Survey Among Italian Associations for Patient Advocacy
Source: JMIR Mhealth Uhealth. 2019 Jan 15;7(1):e10242. doi: 10.2196/10242 (PMC6350090; doi:10.2196/10242)
Supplement: Multimedia Appendix 1 [file mhealth_v7i1e10242_app1.pdf]

Multimedia appendix 1

SURVEY ABOUT DIGITAL INNOVATION: ASSOCIATIONS' OPINIONS
Thank you for taking part in this survey. All your answers are important.<br>The questionnaire is anonymous and data will be analyzed overall

Thinking about the association that you represent:

01 Year of foundation /__/__/__/__/

02 Setting A list was proposed

03 Where is the association's headquarters? A list was proposed

04 In 2016, the association worked activity mainly at level: -just one answer-

Local I__I
Regional (if there is more than one office in the same area) I__I
National (if there are offices in at least five regions) I__I

05 In 2016, the association had its own independent headquarters? I Yes I I No I
the association was open seven days a week? I Yes I I No I
the association had a web-site? I Yes I I No I
the association had a Facebook page? I Yes I I No I
the association had a Twitter account? I Yes I I No I
the association had a YouTube account/channel? I Yes I I No I
the association had a blog? I Yes I I No I

06 In the current association advisory board … -just one answer-
All members are patients or their relatives I__I
Most members are patients I__I
Around half are patients' representatives I__I
A minority are patients' representatives I__I
There are no patients representatives I__I

Thinking about science and technology…

07 In your opinion, 15 years from now, what impact will science and technological innovation have on medical care and health?

I__I Negative Impact I__I No impact I__I Positive Impact I__I I don't know

08 In your opinion, do the following technological innovations have an impact on medical care and health to date?
Health-Apps I Yes I I No I
Wearable devices, such as bracelets, step-counters, smart-watches, etc. I Yes I I No I
Telemedicine services, to monitor and manage the disease at home I Yes I I No I
Online social networks, to communicate, promote health, create networks of patients with the same disease I Yes I I No I

09 In your opinion, will the following technological innovations have an impact about medical care and health at three years?

Health apps	I Yes I I No I
Wearable devices, such as bracelets, step-counters, smart-watches, etc.	I Yes I I No I
Telemedicine services, to monitor and manage the disease at home	I Yes I I No I
Online social networks, to communicate, promote health, create networks of patients with the same disease	I Yes I I No I

10 In your opinion, what is the utility of health-apps and wearable devices on the following aspects of medical care and health?

To be engaged in one's own health	Useful	Not useful	No effect
To improve patient-doctor communication	Useful	Not useful	No effect
To understand one's own health condition	Useful	Not useful	No effect
To reduce costs of healthcare	Useful	Not useful	No effect
To boost compliance	Useful	Not useful	No effect

Now, we are going to consider only health-apps and wearable devices

11 In your opinion, which health-apps and wearable devices are used by members of the healthcare advocacy association you represent?

**Diet app**: to take note of calories, manage diet, receive text message of encouragement, for example to lose weight	Yes	No	I don't know
**Informative app**: to search for information about health or disease	Yes	No	I don't know
**Fitness app**: to monitor physical activity, collect data about running, walking or cycling	Yes	No	I don't know
**Monitoring app**: to control the disease and symptoms through sensors or external devices measuring medical parameters to share them with the doctor, etc…	Yes	No	I don't know
**Therapy app**: to increase compliance, receiving text message that remind you to take pills or complete therapy diaries	Yes	No	I don't know
**Self check app**: to check symptoms	Yes	No	I don't know
**Services app**: to schedule visits/exams or view a medical report	Yes	No	I don't know

**Fitness wearable:** to check physical activity	Yes	No	I don't know
**Weight wearable** : to check weight	Yes	No	I don't know
**Blood pressure wearable**: to check heart rate and blood pressure	Yes	No	I don't know
**Glycaemia wearable**: to check blood sugar	Yes	No	I don't know
**Sleep wearable**: to check rhythm and quality of sleep	Yes	No	I don't know

12 In your opinion, which are the obstacles that members of the healthcare advocacy association you represent, they have in using health-apps and wearable devices?

Technical obstacles, for example not having a suitable device	Yes	No	I don't know
Personal motivations, for example not being able to use them	Yes	No	I don't know
Little faith in usefulness of data recorded	Yes	No	I don't know
Low trust in confidentiality and privacy of data	Yes	No	I don't know
Low trust in accuracy and reliability of data recorded	Yes	No	I don't know
Lack of examples of their usefulness for medical assistance	Yes	No	I don't know

13 In your opinion, thinking about the members of the healthcare advocacy association that you represent, on which health-apps and wearable devices might the developers focus to give more useful tools to improve medical assistance and health?

**Diet app**: to take note of calories, manage diet, receive text message of encouragement, for example to lose weight	Yes	No	I don't know
**Informative app**: to search for information about health or disease	Yes	No	I don't know

**Fitness app**: to monitor physical activity, collect data about running, walking or cycling	Yes	No	I don’t know
**Monitoring app**: to control the disease and symptoms through sensors or external devices measuring medical parameters to share them with the doctor, etc…	Yes	No	I don’t know
**Therapy app**: to increase compliance, receiving text message that remind you to take pills or complete therapy diaries	Yes	No	I don’t know
**Self check app**: to check symptoms	Yes	No	I don’t know
**Services app**: to schedule visits/exams or view a medical report	Yes	No	I don’t know
**Fitness wearable:** to check physical activity	Yes	No	I don’t know
**Weight wearable** : to check weight	Yes	No	I don’t know
**Blood pressure wearable**: to check heart rate and blood pressure	Yes	No	I don’t know
**Glycaemia wearable**: to check blood sugar	Yes	No	I don’t know
**Sleep wearable**: to check rhythm and quality of sleep	Yes	No	I don’t know

14 In your opinion, which negative aspects could be related to a constant adoption of health-apps and/or wearable devices?

Dependence	Yes	No
No privacy	Yes	No
Excessive control of one’s own health	Yes	No
Increasing medicalization, for example through an over-use of drugs, supplements, medical devices or scheduling visits even if they are not needed	Yes	No
Compromising patient-doctor communication	Yes	No

Finally, please answer the following questions about yourself:

Sex /M/ /F/
Age /__/__/

What is your level of education?

- ☐ Elementary school
- ☐ Secondary school
- ☐ High school
- ☐ Degree or higher
- ☐ Other

Do you personally use?

Health apps	I Yes I I No I
Wearable devices	I Yes I I No I

Do you agree to the association you represent being listed among survey responders?

I YesI I No I

If Yes, please write the correct full title of the association: ___________________________
